# Designing Functional and Responsive Molecules with Boronic Acids

**DOI:** 10.1021/acs.accounts.4c00691

**Published:** 2025-02-12

**Authors:** João P. M. António, Inês L. Roque, Fábio M. F. Santos, Pedro M. P. Gois

**Affiliations:** Research Institute for Medicines (iMed.ULisboa), Faculty of Pharmacy, 70880 Universidade de Lisboa, 1649-003 Lisbon, Portugal

## Abstract

Boronic acids (BAs) are one of the most important classes of reagents in modern synthesis, enabling a wide range of powerful transformations that facilitate the formation of key carbon–carbon and carbon–heteroatom bonds. While their success as reagents is well-known, their remarkable potential as building blocks for creating functional molecules is often overlooked.

At the core of BAs’ uniqueness is their ability to form reversible covalent bonds, thanks to the interconversion of the boron atom between its uncharged trigonal planar structure and an anionic sp^3^-hybridized form. This coordination chemistry has paved the way for exciting developments in fields such as medicinal chemistry and chemical biology. In recent years, BAs have been used to create a wide variety of materials, including small-molecule drugs, bioconjugates, drug delivery vehicles, polymeric nanomaterials, sensors, and even photosensitizers. What makes this strategy particularly unique is the structural diversity that can be achieved by functionalizing the BA coordination sphere, along with the possibility of incorporating stimuli-responsive mechanisms. This reactivity is further enhanced by the well-known oxidation of BAs in the presence of reactive oxygen species (ROS).

A detailed understanding of the mechanisms governing the dynamic nature of BAs enables the engineering of sophisticated materials that can respond to specific molecular stimuli, such as changes in pH, carbohydrate or glutathione concentrations, and hydrogen peroxide. These stimuli are often key indicators of diseases such as cancer, inflammation, and neurodegeneration, placing BAs at the forefront of tools for designing materials that can potentially influence the mechanisms behind these diseases.

In this Account, we draw on our group’s expertise to explore the exciting potential of BAs in the design of functional materials. The focus is on the response of different boron complexes to biologically relevant stimuli. We describe the preparation of boronated esters (BEs), BA–salicylhydroxamic acid (BA–SHA) complexes, iminoboronates, diazaborines, and boronated thiazolidines and discuss how these chemotypes respond to disease-relevant triggers. Given the growing importance of using external stimuli to control the efficacy of modern drugs, we also explore how some of these compounds respond to specific chemicals. While this Account is not meant to be an exhaustive survey of every example of BA stimulus-responsiveness, we aim to integrate existing chemotypes and their chemical triggers. Our goal is to provide an overview of the mechanisms enabled by BAs for designing functional materials that could one day lead to innovative therapeutic options for human diseases.

## Graphical abstract

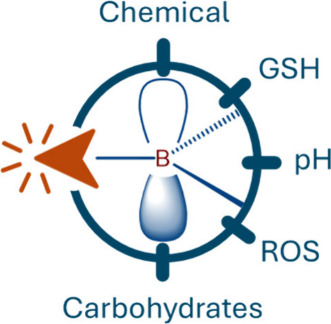

## Key References

-

Cal, P. M. S. D. ; Vicente, J. B. ; Pires, E. ; Coelho, A. V. ; Veiros, L. F. ; Cordeiro, C. ; Gois, P. M. P. Iminoboronates: A New Strategy for Reversible Protein Modification. J. Am. Chem. Soc. 2012, 134 (24), 10299–10305.22642715 10.1021/ja303436y^1^ *We demonstrated for the first time that iminoboronates are a suitable technology to modify lysines in physiological conditions.*
-

Faustino, H. ; Silva, M. J. S. A. ; Veiros, L. F. ; Bernardes, G. J. L. ; Gois, P. M. P. Iminoboronates Are Efficient Intermediates for Selective, Rapid and Reversible N-Terminal Cysteine Functionalisation. Chem. Sci. 2016, 7 (8), 5052–5058 30155155 10.1039/c6sc01520dPMC6018717.^2^ *We demonstrated that 2-carbonyl phenylboronic acids react extremely fast with N-terminal cysteines and explored this methodology to create controlled dual-modified bioconjugates.*
-

Santos, F. M. F. ; Matos, A. I. ; Ventura, A. E. ; Gonçalves, J. ; Veiros, L. F. ; Florindo, H. F. ; Gois, P. M. P. Modular Assembly of Reversible Multivalent Cancer-Cell-Targeting Drug Conjugates. Angew. Chem., Int. Ed. 2017, 56 (32), 9346–9350 10.1002/anie.20170349228594469.^3^ *In this work, we developed a boronated modular platform to generate complex multivalent constructs. These constructs, which are responsive to glutathione, were used in the preparation of tumor-targeting drug conjugates.*
-

António, J. P. M. ; Carvalho, J. I. ; André, A. S. ; Dias, J. N. R. ; Aguiar, S. I. ; Faustino, H. ; Lopes, R. M. R. M. ; Veiros, L. F. ; Bernardes, G. J. L. ; da Silva, F. A. ; Gois, P. M. P. Diazaborines Are a Versatile Platform to Develop ROS-Responsive Antibody Drug Conjugates. Angew. Chem., Int. Ed. 2021, 60 (49), 25914–25921 10.1002/anie.20210983534741376.^4^ *Despite being very stable in a broad range of pH and plasma, we demonstrated in this work that diazaborines are responsive to reactive oxygen species. Using this knowledge, we designed the first reported ROS-responsive antibody–drug conjugate.*

## 1 Introduction

Recent advances in synthetic chemistry and borylation methods have made the preparation of organoboron compounds such as boronic acids (BAs) simple and economically accessible, revitalizing this class of compounds.^5^ Since then, BAs and their corresponding esters (BEs) have become a central class of reagents in modern synthesis, enabling powerful transformations that create essential C–C and C–heteroatom bonds.^6^ While the remarkable utility of BAs as reagents is widely recognized, their potential as fundamental building blocks has traditionally been somewhat overlooked. However, over the last 20 years, a newfound appreciation for the unique properties of this functional group has firmly established BAs as essential components in the creation of functional molecules across several areas, including medicinal chemistry,^7^ materials science,^8^ and chemical biology.^9,10^

While this Account focuses on stimuli-responsive BA complexes, it is worth noting that organoboron compounds comprise other diverse structures including borinic acids and hemiboronic acids, each displaying unique properties. Both boronic acids and hemiboronic acids have been widely use as catalysts for challenging organic reactions,^11,12^ and recently, boronic acids were shown to be efficiently reactive oxygen species (ROS)-responsive, highlighting the potential of these two classes as future stimuli-responsive warheads.^13^

BAs have a vacant *p* orbital centered on the boron atom, which allows the formation of reversible covalent bonds with various Lewis bases.^14^ In particular, their mild Lewis acidity allows BAs to exist in their uncharged state under physiological conditions, making them suitable for reversible complexation of nucleophiles at protease active sites. This property has attracted the interest of the medicinal chemistry community, leading to the use of BAs as transition state analogues in the design of protein inhibitors.^10,15^

The coordination chemistry of BAs is both rich and dynamic, involving the interconversion of the central boron atom between an uncharged trigonal planar structure and an anionic sp^3^-hybridized borate species. The strategic selection of ligands allows the creation of diverse boron complexes with distinctive molecular architectures and properties. This dynamic coordination profile has been invaluable in the design of novel materials for drug delivery, sensing and bioimaging applications.^16^ In addition to their characteristic reactivity, the biocompatibility and mild Lewis acidity of BAs allow the precise modification of specific functional groups within complex systems. Specifically, BAs have been used in bioconjugation reactions targeting solvent-exposed amino acids on peptides and proteins and have played a crucial role in the synthesis of well-defined bioconjugates.^17,18^ In all these applications, the boron coordination sphere either serves as a template for the dynamic binding of ligands or exploits the reactivity of neighboring functional groups, highlighting the unique potential of this functional group as a construction tool. However, this remarkable utility is not the only feature offered by BAs. The stability of BA-based reversible covalent bonds is highly dependent on microenvironmental factors such as pH and the concentration of endogenous molecules such as carbohydrates and reduced glutathione (GSH). In addition, aromatic BAs are known to undergo rapid oxidation to phenols in the presence of ROS. This unique reactivity profile grants BAs an additional level of utility in designing materials equipped with mechanisms to respond to specific chemical stimuli. This responsiveness is particularly important in the discovery and development of new drugs for complex diseases such as cancer, neurodegeneration, and inflammation, where these chemical triggers often serve as key molecular features. Therefore, building on our group’s expertise, in this Account we explore how BAs can serve as a highly versatile and well-defined molecular platform, uniquely equipped to respond to a range of biologically relevant stimuli like pH, carbohydrates, GSH, ROS and chemical inducers. Importantly, this Account stands out by categorizing boron complexes based on specific biological stimuli, providing a clear and application-oriented perspective. This structure helps readers identify suitable boron structures for their desired stimulus-response system, facilitating a targeted design for various applications.

## 2 pH Responsiveness

The human body maintains a slightly alkaline extracellular pH range of approximately 7.3–7.4, while intracellular pH levels vary between pH 4.7 in lysosomes and pH 7.2 in the cytosol. This tight pH regulation is critical for optimal cellular function, and deviations of more than 0.05 units can lead to acidosis, observed in conditions such as diabetes, cancer, and neuroinflammation.^19^ In recent years, the potential of BEs, boronic acid–salicylhydroxamic Acid (BA–SHA) complexes, and iminoboronates for reversible covalent reactions in pH-catalyzed hydrolysis has enabled the design of numerous pH-responsive constructs, polymeric carriers, and nanoparticles with applications across various fields, including medicinal chemistry and chemical biology.

### 2.1 Boronate Esters

In aqueous medium, BAs and their respective BEs coexist in an equilibrium that is governed by an acid dissociation constant (*K*_a_) or *K*_d_ (=1/*K*_a_). The *K*_a_ characterizes the binding affinity of a BA (or BE) to an alcohol substrate and is strongly dependent on the pH,^20^ as both the BA and BE can exist in a mixture of neutral and anionic forms, established by their individual p*K*_a_ values.^21^ At neutral or slightly acidic pH, BAs exist predominantly in their neutral form (sp^2^ hybridization) with pH_medium_ < p*K*_a_ of the boron species. However, under basic conditions (pH_medium_ > p*K*_a_ of the boron species), they form boronate anions, which upon reacting with alcohols or diols, lead to the formation of BEs (sp^3^ hybridization) (Figure 1).^15^ In general, the p*K*_a_ values of BAs/BEs are in the range of 4.0 to 10.5.^22^ Therefore, careful tuning of the BA structure is both possible and often necessary to achieve the ideal outcome for each desired application. More detailed discussions on the boronate ester formation have been reviewed elsewhere.^20^

**Figure 1:**
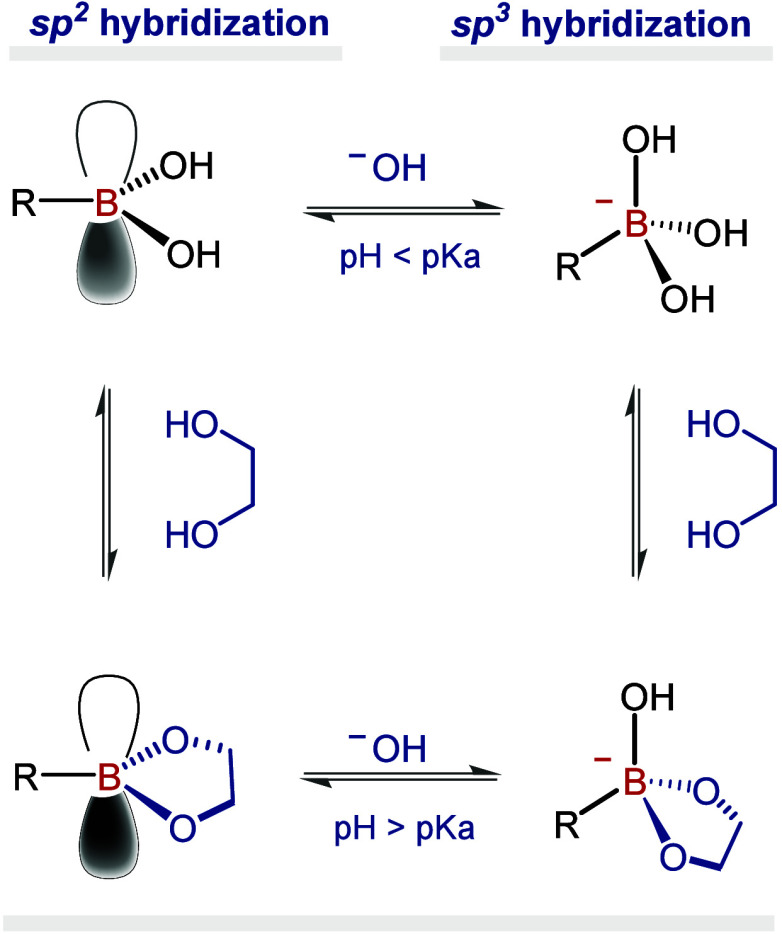
Influence of pH in the formation equilibrium of BEs in aqueous solution.

The significant influence of pH on the formation and dissociation of BEs has triggered the development of constructs capable of responding to biological pH changes, particularly acidic environments. This approach typically involves the incorporation of BEs into polymeric or supramolecular structures, which then undergo hydrolysis in acidic conditions, leading to a predetermined effect such as payload release. This strategy has demonstrated significant success in designing pH-responsive materials and has been extensively reviewed elsewhere.^23^

### 2.2 Boronic Acid–Salicylhydroxamic Acid Complexes

Owing to its empty *p* orbital, BAs can establish reversible covalent ligations with Lewis bases, such as diols, to generate BEs. However, this complexation is not limited to oxygen-based Lewis bases. In fact, BAs are also able to bind strongly to SHAs under physiological conditions. Wiley and co-workers initially discovered that SHA forms a stable complex across a wide range of physiological pH levels, which readily hydrolyzes under acidic conditions.^24^ Using ^11^B NMR, they demonstrated that, at neutral and alkaline pH, the boron within the complex displays tetrahedral sp^3^ hybridization, while at acidic pH, a mix of sp^2^, sp^3^, and BA signals is observed, suggesting hydrolysis. They also showed a preference for the formation of a six-membered ring over a five-membered ring (Figure 2). The authors successfully applied this concept to protein immobilization and purification. More recently, Weil and co-workers studied the responsiveness of the BA–SHA ligation in a series of works aimed at the modulation, activation, and delivery of large proteins. First, Weil and co-workers designed a new class of hybrid zymogens by attaching protective poly­(amide)­amino dendrimers to proteases, rendering them inactive until they enter an acidic lysosomal environment where the dendrimers dissociate, reactivating the enzymes (Figure 3).^25^ In addition to these important studies, Weil’s Group further explored the PBA-SHA dynamic response to acidic pH in the pH-responsive PEGylation to modulate protein pharmacokinetics,^26^ the development of smart hydrogels for targeted enzyme delivery,^27^ and the controlled supramolecular assembly of peptides inside cells.^28^ More recently, Thayumanavan and co-workers developed a new generation of responsive protein–antibody conjugates (PACs) using a spherical polymer brush-based nanoparticle scaffold.^29^

**Figure 2:**
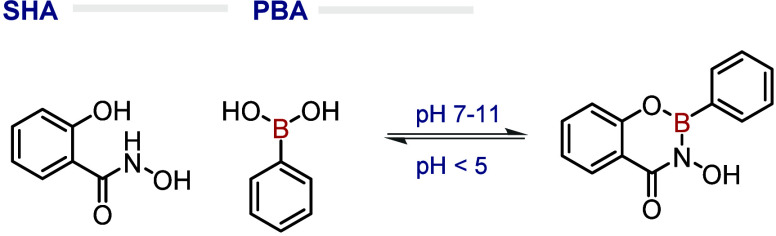
Assembly of a complex between SHA and PBA at pH between 7 and 11 and its disruption below pH 5.

**Figure 3:**
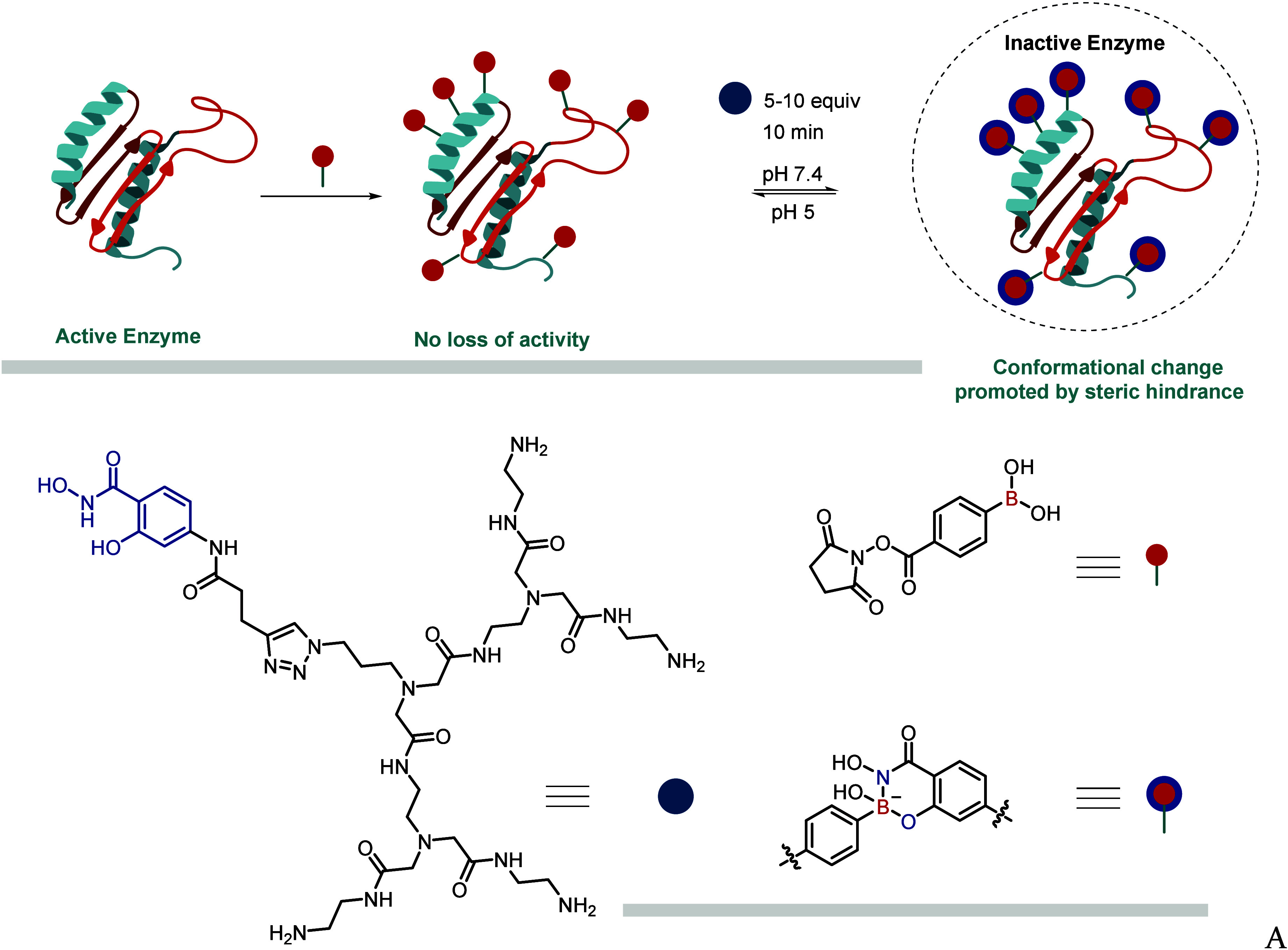
BA–SHA response to the modulation and activation of large proteins depending on pH. In a physiologic environment (pH 7.4) the proteases are inactive due to the protective poly­(amide)­amino dendrimers. These dendrimers will dissociate in an acidic environment (pH 5) restoring the enzyme properties.

### 2.3 Iminoboronates

Since their discovery in 1968 and the subsequent work by the James and Nitschke groups on the resolution of chiral amines, iminoboronates, prepared through the condensation of *o*-carbonyl phenylboronic acids (PBAs) and amines, have found numerous applications. In 2012, both our group^1^ and the Yatsimirsky group^30^ independently reported that, in aqueous media, 2-carbonyl PBAs efficiently react with exposed amino groups of peptides, proteins, and amino sugars at low-millimolar concentrations.

While the formation of imines under physiological conditions is thermodynamically unfavorable, iminoboronates exhibit enhanced hydrolytic stability. Density functional theory (DFT) calculations performed on this system suggest a mechanism in which the BA acts as an intramolecular Lewis acid, facilitating the dehydration step that leads to imine formation, and subsequently stabilizing the iminoboronate through B–N bond formation^1^ (Figure 4A). However, more recent studies indicate that, in methanol, the thermodynamic stability of this function is attributed to ion-pair interactions resulting from solvent insertion (Figure 4B). Our initial studies on iminoboronates also revealed that these reactions exhibit fast kinetics and relatively high equilibrium constants, with a reaction profile that remained mostly unchanged over a week and within a pH range of 6–9. Regardless of the exact mechanism governing their stability in aqueous environments, this technology has achieved considerable success in the bioconjugation of peptides and proteins^1,18^ (Figure 4C), design of enzyme inhibitors,^31^ pH-controlled peptide cyclization,^32,33^ studying ion current in transmembrane ion channels,^34^ phage display technology^35,36^ and in the construction of hybrid materials.^37^

**Figure 4:**
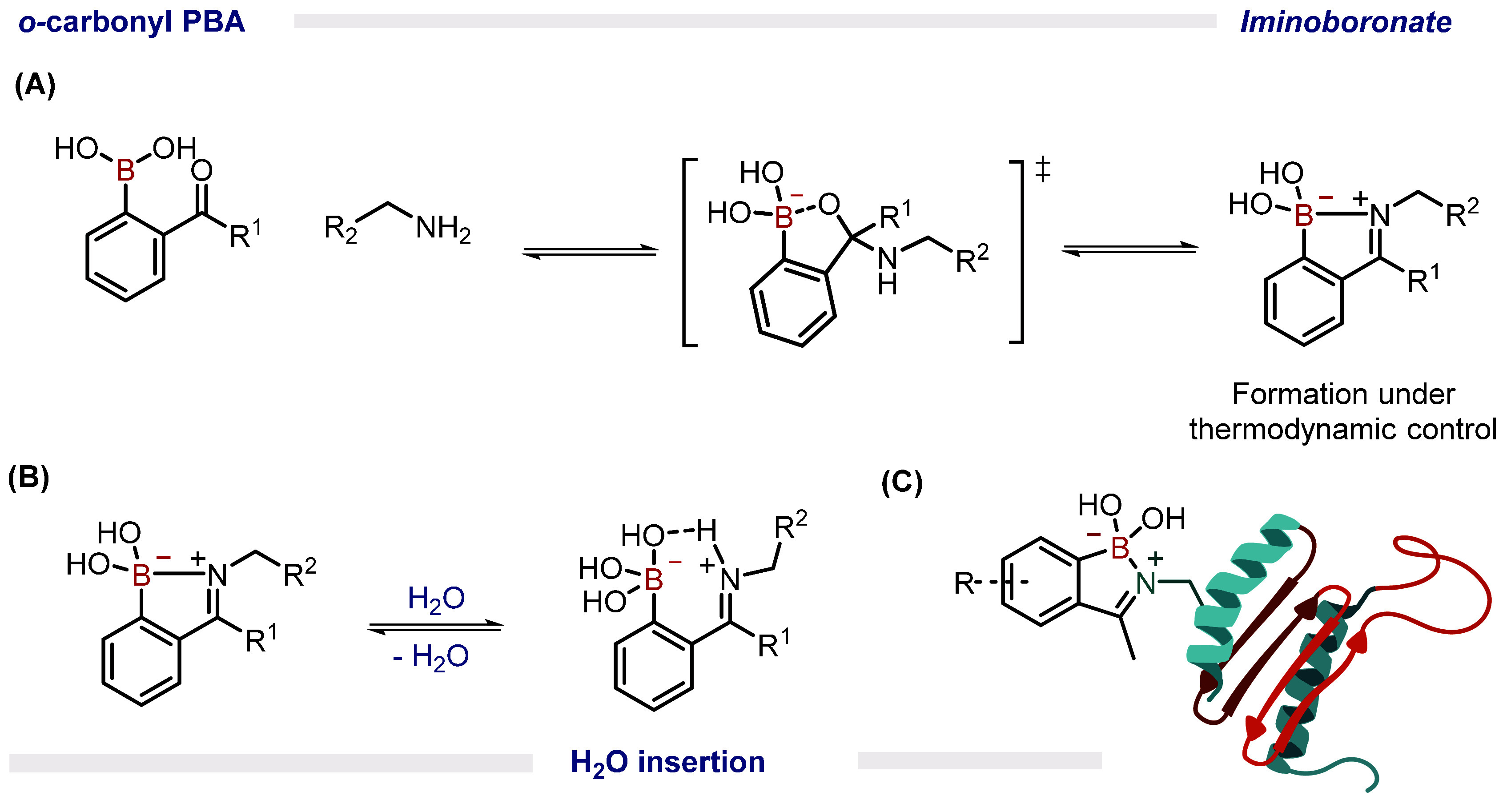
(A) Formation of iminoboronates by reaction of *o*-carbonyl PBA with distinct amino groups in (B) aqueous media. (C) Exposed amino groups of different peptides or proteins can also be modified to insert an iminoboronate group.

In a clear demonstration of the usefulness of this technology, Gao and co-workers targeted abundant and accessible amino-phospholipids, such as phosphatidylethanolamine (PE) and lysylphosphatidylglycerol (Lys-PG), in bacterial cell walls using a fluorophore-labeled 2-formylphenylboronic acid (2-FPBA).^38^ This straightforward method allowed for the installation of iminoboronates and the successful staining of Gram-positive bacteria (*B. subtilis* and *S. aureus*) in the presence of Gram-negative bacteria (*E. coli*) and mammalian cells (Jurkat) (Figure 5).

**Figure 5:**
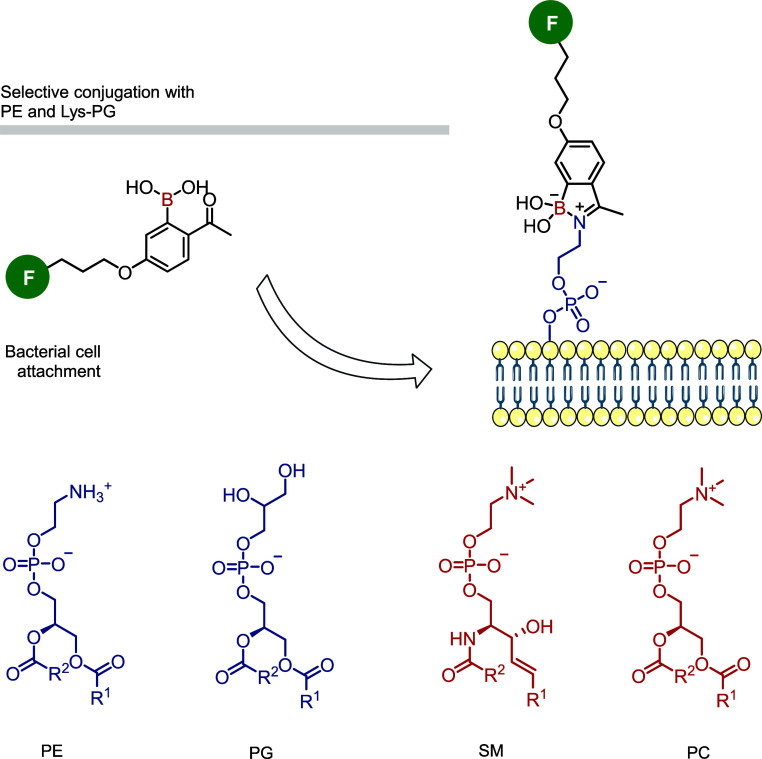
Conjugation of different amino-phospholipids (PE, PG, SM, PC), present in bacterial wall, with fluorophore-labeled 2-FPBA to form stable iminoboronates responsible for only staining Gram-positive bacteria.

Additionally, the incorporation of iminoboronates as cross-linkers for supramolecular structures has proven to be a successful strategy, offering protection from hydrolysis at neutral pH while allowing hydrolysis of the construct in more acidic conditions. This approach has been used, for example, in the construction of responsive iminoboronate-chitosan hydrogels with strong antifungal activity,^39^ as well as humidity-sensitive, self-healing polymers.^37^

Despite these advances, the use of iminoboronates in well-defined pH linkers remained elusive. Aware of this limitation, Hall and co-workers reported that nopoldiol derivatives and 2-methyl-5-carboxymethylphenylboronic acid establish a fast and dynamic ligation (*k*_ON_ = 7.7 ± 0.5 M^–1^ s^–1^, *K*_eq_ = 1.2 × 10^5^ M^–1^).^40,41^ This reaction mode was employed in the design of a synergistic system incorporating thiosemicarbazide-functionalized nopoldiol and *o*-acetyl arylboronic acid, resulting in the rapid and irreversible formation of a boronate/iminoboronate dual adduct with a rate constant of 9.0 M^–1^ s^–1^ (Figure 6A).^40^ This stability profile proved essential for the labeling of live HEK293T cells (Figure 6B) and the *in vivo* capture of systemically circulating fluorescent nopoldiol reagents by arylboronate motifs anchored in the extracellular matrix (Figure 6C).^42^ A similar approach was pursued by Kanai and co-workers which used the boronate-nopoldiol complexation to acylate lysine residues.^43^

**Figure 6:**
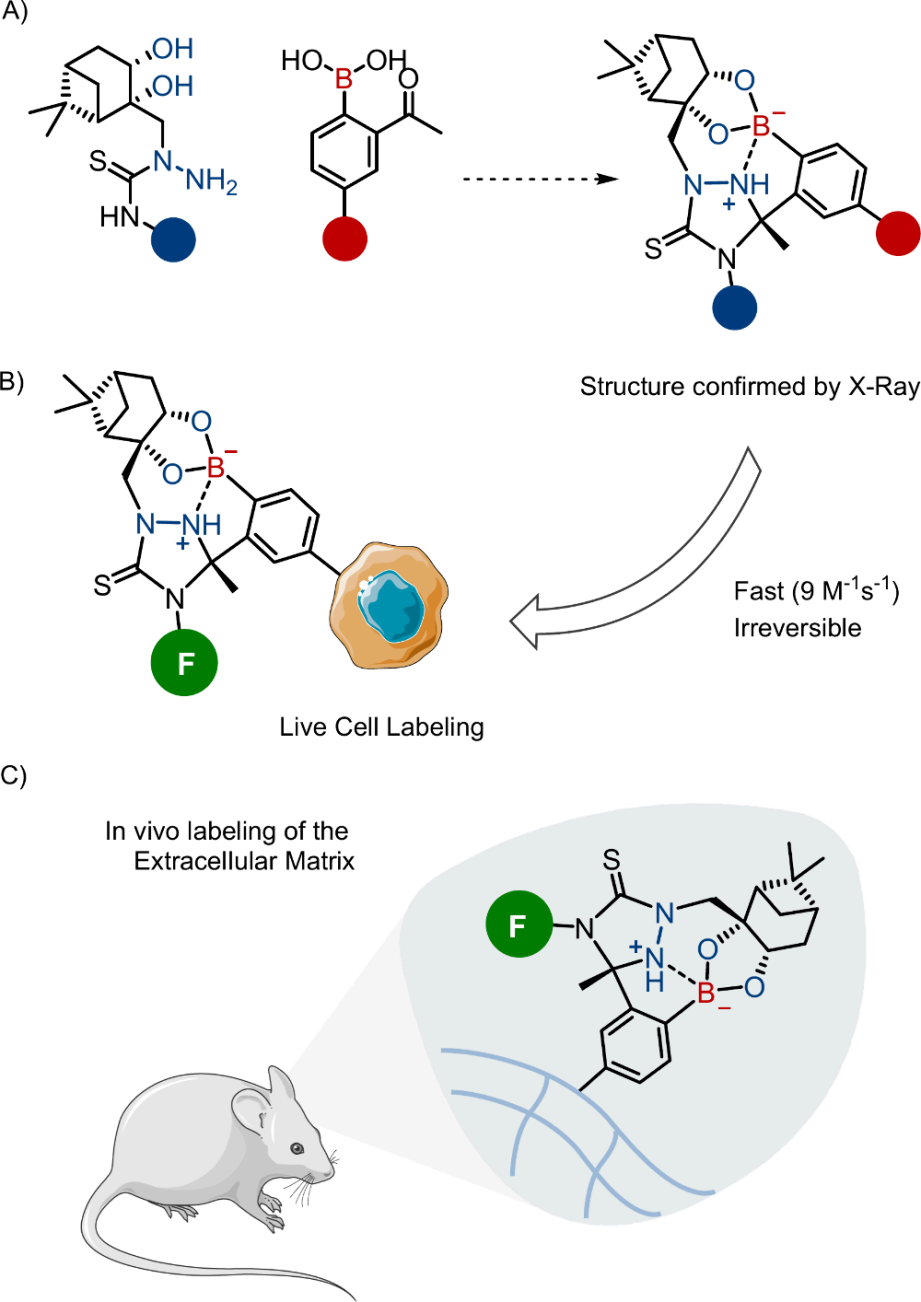
(A) Fast and dynamic boronate/iminoboronate dual adduct establish between nopoldiol derivatives and 2-FPBA. (B, C) Fluorescence can be added to the molecule for (B) live cell or (C) in vivo labeling.

Inspired by the work of Anslyn, Bull, James, and Hall on the stabilization of imines through the formation of BEs,^44–47^ we considered the possibility of stabilizing the iminoboronate by forming an additional intramolecular B–O bond.^48^ This class of iminoboronates was synthesized by reacting 2-FPBA or 2-acetylphenylboronic acid (2-APBA) with 2-aminomethyl phenols, resulting in a particularly stable cyclic structure. This core demonstrated good stability at pH 7.4 and in human plasma, with less than 10% and 20% degradation within 48 h, respectively, while remaining susceptible to hydrolysis under acidic pH conditions (*t*_1/2_ = 5.8 h in PBS at pH 4.5). The versatility of this platform was further demonstrated in the construction of a folic acid conjugate that delivered a fluorescent probe to human breast cancer cells. The fluorescent probe exhibited quenched fluorescence upon incorporation into the *N,O*-iminoboronate core, which was restored upon folic acid-mediated internalization of the conjugate in MDA-MB-231 human breast cancer cells and subsequent hydrolysis of the *N,O*-iminoboronate (Figure 7).

**Figure 7:**
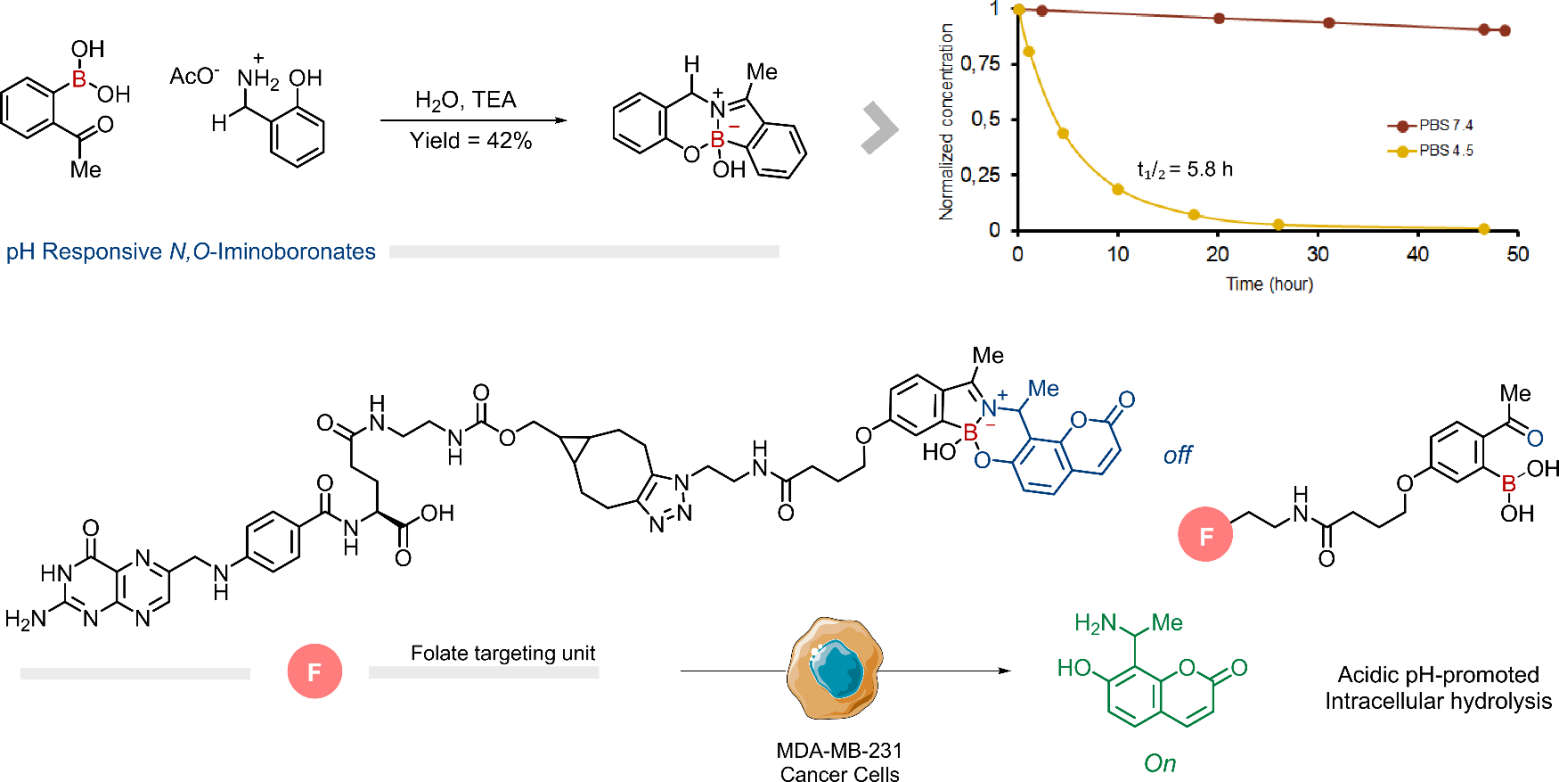
Stabilized iminoboronates with aminophenols and 2-APBA. Below is the folate derivative of the *N*,*O*-iminoboronate with an aminocoumarin that presents fluorescence when released at acidic pH.

## 3 Carbohydrate Responsiveness

Carbohydrates typically exhibit 1,2- or 1,3-diol functionalities, which interact with BAs to form five- or six-membered cyclic BE structures in aqueous media.^21^ The reaction of BAs with saccharides, polysaccharides, or glycoproteins often occurs at submillimolar concentrations; therefore, BAs have found considerable success as warheads for designing sensors for carbohydrates.^49,50^ The binding strength of BAs to carbohydrates is determined not only by the stereoelectronic properties of the BA, but also by the relative position of the hydroxyl groups, allowing the affinity of structurally similar saccharides to be distinguished by a single BA function.^15^

### 3.1 Boronate Esters

The unique coordination profile of BAs with carbohydrates has made them a preferred strategy for designing drug delivery materials that respond to specific endogenous or exogenous carbohydrates. This topic has been extensively reviewed elsewhere.^51^ Therefore, here we present just an example of this mechanism applied in the construction of responsive polymers. Chou et al. designed a glucose-sensitive system for insulin delivery, where insulin was linked to albumin using an alkyl-lysine chain capped with a PBA-based glucose sensor.^52^ In diabetic mice, this system successfully regulated blood glucose levels, with a single dose maintaining glucose control for 12 h across multiple glucose tolerance tests. The responsiveness of this technology offers an exciting strategy for treating type II diabetes patients, allowing for the availability of monomeric insulin (the active form) as a direct response to an increased concentration of carbohydrate in the serum.

### 3.2 Iminoboronates

As mentioned previously, iminoboronates are dynamic functions that are particularly sensitive to the concentrations of diols like fructose and dopamine. This reactivity has been explored by our group to design a responsive insulin conjugate. Covalent modification of peptides and proteins with polyethylene glycol (PEG) chains is a widely used strategy to prevent degradation by proteolytic enzymes and to enhance their stability and biodistribution. This approach has been actively pursued in the development of oral insulin. Consequently, we prepared a PEGylated BA for the modification of insulin, which was shown to break down in the presence of fructose. Although the reaction was not complete, the concentration of PEGylated constructs in solution decreased significantly under these conditions, regenerating free insulin.^18^ (Figure 8).

**Figure 8:**
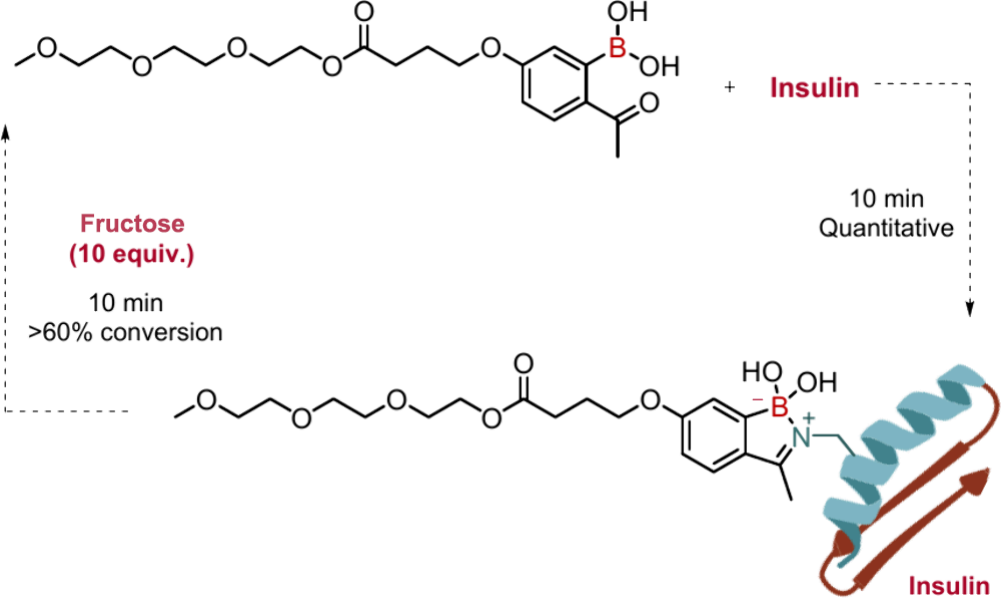
Installation of a PEG chain on a 2-acetylbenzeneboronic acid core and further reaction with insulin. The presence of fructose prompted the insulin construct hydrolysis into to free insulin and the correspondent PEGylated boronic acid.

## 4 Glutathione Responsiveness

GSH, a tripeptide composed of glutamate, cysteine, and glycine, is a cellular antioxidant that plays a crucial role in cancer due to its involvement in redox regulation, detoxification, and the maintenance of cellular homeostasis.^53^ Therefore, designing boron complexes that respond to GSH concentration is an appealing strategy for developing responsive systems to deliver drugs to cancer cells or activate probes, as recently described by Cao and co-workers.^54^

### 4.1 Iminoboronates

The functionalization of exposed amino groups in peptides and proteins can be successfully achieved using 2-APBA, as demonstrated by the modification of the neuropeptide somatostatin and proteins such as lysozyme, cytochrome *c*, ribonuclease A, and myoglobin.^1^ During these studies, we observed that these iminoboronates also hydrolyze in the presence of GSH. In cancer cells, GSH concentrations are severalfold higher than in normal cells (millimolar range), and this hallmark of cancer has been extensively targeted by various technologies to promote drug release. Therefore, we utilized iminoboronate technology to assemble responsive cancer cell-targeting conjugates. First, human nonsmall cell lung cancer cells overexpressing folate receptors were successfully labeled using a folate derivative linked to a fluorescent probe.^55^ Similarly, iminoboronate technology was used to prepare a folate-paclitaxel small-molecule drug conjugate that exhibited significant cytotoxicity against NCI-H460 cancer cells (IC_50_ = 20.7 nM)^18^ (Figure 9). The mechanism through which GSH promotes the cleavage of the iminoboronate is still unknown and has not been studied in detail. However, as can be seen in the previous sections, iminoboronates also display reduced stability in acidic media and in the presence of certain carbohydrates. Therefore, it is likely that the observed reversibility is not specific to GSH and may occur due to other indirect mechanisms (dilution/acidification of the sample media). Therefore, while iminoboronates appear to display some level of reversibility in the presence of GSH, their general low stability strongly deters their usage beyond certain dynamic applications.

**Figure 9:**
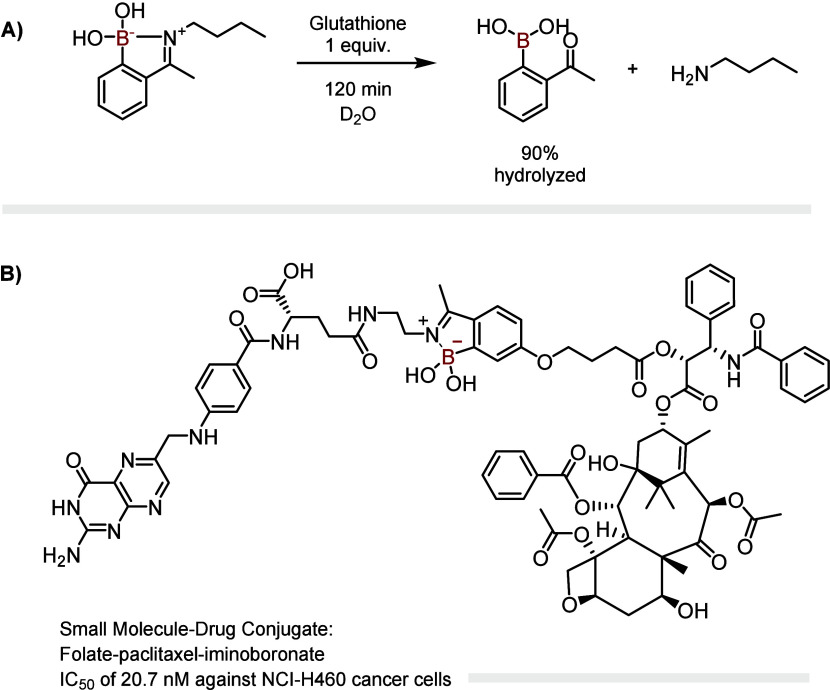
(A) Iminoboronates are reversible in the presence of GSH; (B) GSH-sensitive iminoboronates were used in the preparation of a cytotoxic bioconjugate.

### 4.2 Boron Hotspots

Once we established that iminoboronates featuring an additional B–O bond exhibited improved physiological stability, we explored the possibility of using a similar principle to design a synthetic boron hot spot for selective and reversible protein modification. In this method, a maleimide-functionalized 3-hydroxyquinoline (3-HQ) derivative is attached to an N-terminal cysteine on the protein. The 3-HQ acts as a ’boron hot spot,’ directing the iminoboronate formation to the N-terminus. This boron hot spot was shown to bind strongly to phenylboronic acid (PBA) (*K*_a_ = 699 M^–1^), through boronate formation, and to react with 2-FPBA (*K*_a_ = 58,128 M^–1^), to generate an iminoboronate adduct that remained stable in buffer at pH 7 for up to 4 weeks. However, since its stability was significantly reduced in the presence of GSH, this motif was then used in the design of a target for the 67LR receptor to selectively deliver cargo to HT-29 cancer cells (Figure 10).^56^

**Figure 10:**
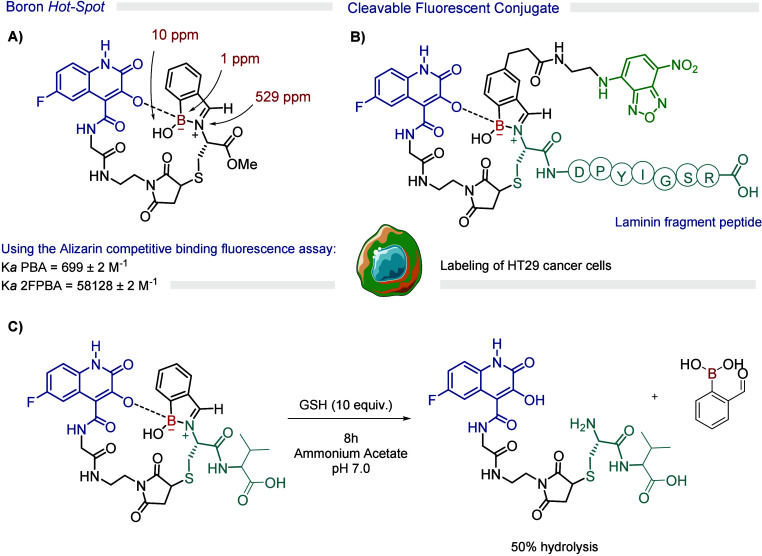
Proposed strategy to site-selectively install an iminoboronate in a peptide chain using a boron hot spot (BHS) strategy.

### 4.3 Tridentate Schiff Base Boron Complexes

Boronic acids are well-known reagents in various multicomponent protocols, such as the Petasis–borono-Mannich reaction.^57^ However, a judicious choice of ligands, such as tridentate Schiff bases, can generate an array of structurally diverse boron complexes.^58,59^ In 2017, we conceived that if these modular complexes exhibited specific mechanisms to respond to disease-associated chemical stimuli, they would be particularly useful as a platform for designing linkers for the construction of antibody– or small-molecule–drug conjugates. To test this hypothesis, we prepared several model boron complexes with tridentate Schiff bases in yields of up to 90% and assessed their stability under biological conditions. The most promising scaffold, derived from phenylboronic acid, 2-aminophenol, and 2-hydroxy-4-methoxyacetophenone, showed a half-life of 39.8 h at pH 7.4, with a similar stability profile at lysosomal pH 4.8 (*t*_1/2_ = 38 h) and in human plasma (*t*_1/2_ = 39.6 h). However, stability was significantly lower in the presence of GSH (10 equiv; *t*_1/2_ = 32.8 h). Given its hydrolytic stability and GSH-responsiveness, we used this framework to develop targeted drug conjugates (TDCs) incorporating PEG chains for increased hydrophilicity, folic acid for targeting cancer cells that overexpress folate receptors, and bortezomib (BTZ), an FDA-approved drug with a poor pharmacokinetic profile. Conjugates containing one or two folic acid groups and bortezomib were tested against the folate receptor-positive (FR+) MDA-MB-231 breast cancer cell line, yielding IC_50_ values of 67.5 nM (monovalent conjugate), 62.0 nM (divalent conjugate), and 14.2 nM (BTZ) (Figure 11).^3^

**Figure 11:**
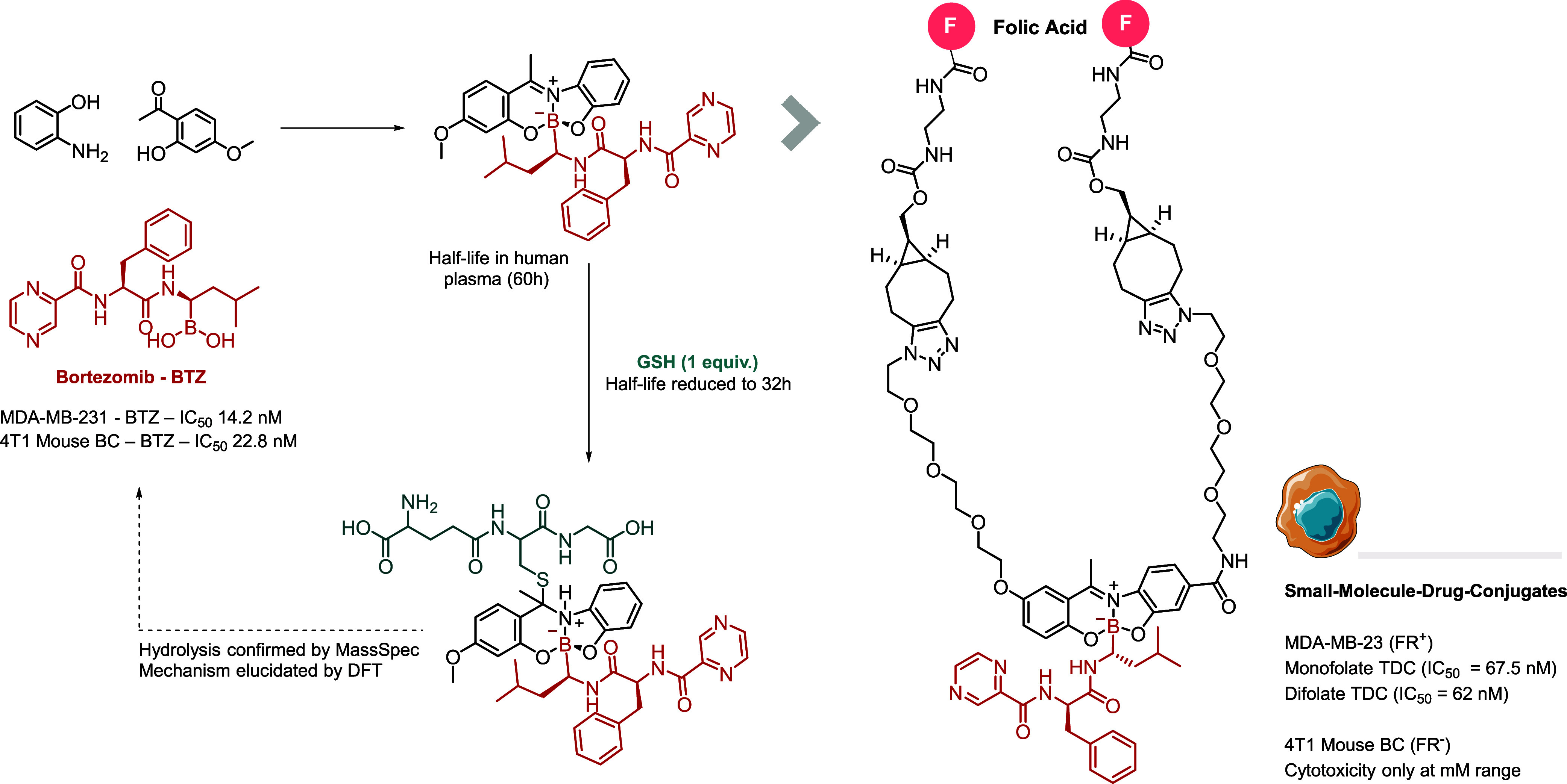
GSH-responsive disassembly of boron-based TDCs containing the cytotoxic drug BTZ.

## 5 ROS Responsiveness

In healthy cells, a complex biological system maintains low and regulated levels of ROS, which are crucial players in cell signaling mechanisms, homeostasis, and immunity. While low levels of ROS play an essential role in cell signaling and the regulation of physiological processes, excessive ROS production can overwhelm cellular antioxidant defenses, leading to oxidative damage to proteins, lipids, and DNA. This nonspecific damage triggers cellular dysfunction and disrupts processes that are key to diseases such as cancer, inflammation, ischemia/reperfusion injury, Alzheimer’s disease, and Parkinson’s disease. As a result, ROS has emerged in recent years as a promising target for many therapies.

### 5.1 Boronic Acids

H_2_O_2_ is the most stable of the endogenously produced ROS. It is well established that, at physiological pH, the hydroperoxide ion (HOO^–^) can attack the electron-deficient boron center to form a tetrahedral boronate, which, upon a thermodynamically favored B–O 1,2-shift, generates an intermediate that spontaneously hydrolyzes to an alcohol or phenol. Aromatic boronic acids react rapidly with H_2_O_2_ (10 M^–1^ s^–1^), and this process is even faster with peroxynitrite (ONOO^–^) (10^6^ M^–1^ s^–1^). Therefore, this trigger has been extensively used in the activation of fluorescent probes^60^ (Figure 12A), prodrugs^61^ (Figure 12B), and in controlling the structure of different materials.

**Figure 12:**
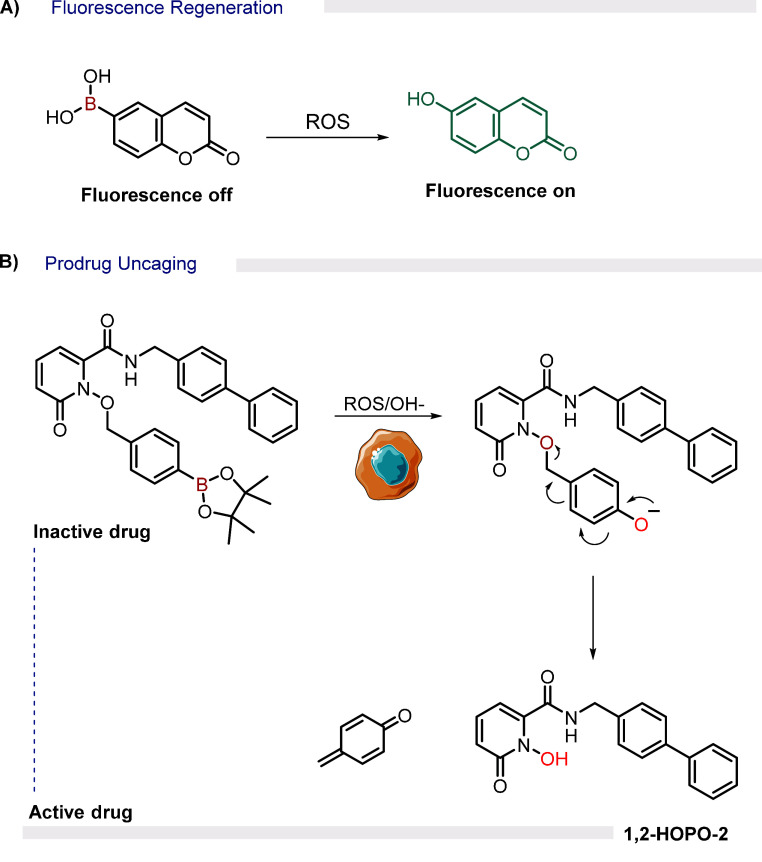
Selected examples of BAs used as ROS-responsive warheads for (A) fluorescence regeneration^62^ and (B) prodrug uncaging.^63^

Labruère and co-workers described a novel prodrug strategy using a vinyl boronic acid that reacted rapidly with both hydrogen peroxide and peroxynitrite (*k*_1_ = 130.0 and 1.8 × 10^4^ M^–1^ s^–1^, respectively) and cyclized intramolecularly to generate a cytotoxic phenanthridine.^64^ The authors demonstrated the feasibility of their approach using both *in vitro* and *in vivo* models, showing efficient activation of the prodrugs within KB tumor spheroids and subsequent cytotoxic effects on cancer cells (Figure 13). However, due to their inherently low circulatory stability and promiscuous binding profile with endogenous Lewis bases, boronic acids (BAs) often require incorporation into supramolecular structures that protect them from oxidation and off-target reactivity. Despite these limitations, there are numerous examples of ROS-responsive drug delivery systems using BAs, which have been extensively reviewed in the literature.^65^ However, we were particularly interested in applying this concept to well-defined structures with improved stability, eliminating the need for supramolecular protection

**Figure 13:**
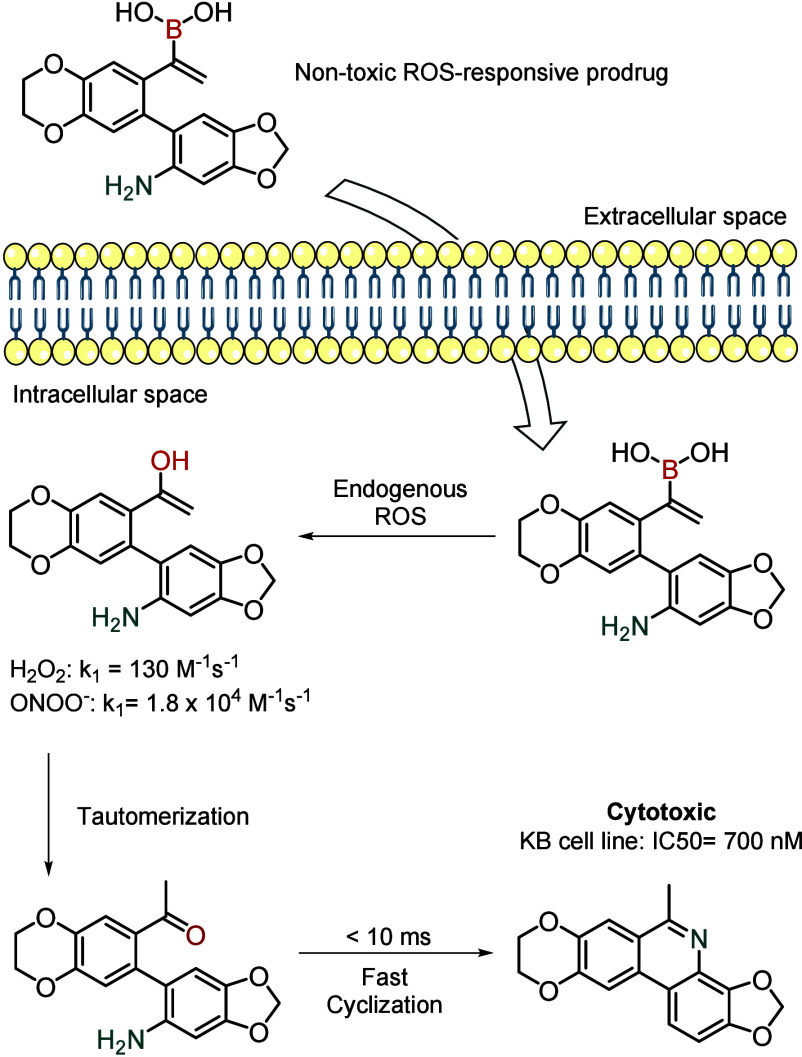
Oxidation of a nontoxic boronic acid inside tumor cells leads to an intramolecular cyclization, generating a toxic compound.

### 5.2 Diazaborines

An important advance in this field was reported by Bane and co-workers, who demonstrated that hydrazones formed from 2-FPBA and 4-hydrazinylbenzoic acid can undergo a second internal dehydration step to form a stable boron–nitrogen heterocycle known as 1,2-dihydro-1-hydroxy-2,3,1-benzodiazaborine^66^ (Figure 14A). This reaction is exceptionally fast in aqueous environments.^67^ Since then, various groups have successfully used diazaborines (DABs) in protein modification^68,69^ and bacteria labeling.^70^ Our group has recently discovered that, despite the partial occupation of the vacant boron orbital, these compounds exhibit reactivity with ROS to generate a salicylhydrazone scaffold. In this study, we present the synthesis of a ROS-responsive antibody–drug conjugate (ADC).^4^ First, we conducted a detailed study of the stability and formation kinetics of eight different DABs with various hydrazines and boronic acids (Figure 14B,C). After selecting 2-FPBA and benzylhydrazine as the ideal pair, we demonstrated the oxidation of the DAB in the presence of 100 and 10 equiv of hydrogen peroxide (*k* = 0.422 M^–1^ s^–1^, *t*_1/2_ = 15 min and *k* = 0.102 M^–1^ s^–1^, *t*_1/2_ = 210 min, respectively) (Figure 14D). Importantly, the oxidation mechanism remains functional in the presence of equimolar concentrations of GSH. After confirming the mechanism and performing DFT calculations to support it, we proceeded to prepare the ROS-responsive ADC. Consequently, we developed and evaluated two different self-immolative modules that promoted the release of a cytotoxic SN38 payload upon reaction with tumor-associated ROS (Figure 15). Following conjugation with a B-cell lymphoma-targeting single-chain nanobody, our ADC was evaluated against the CLBL-1 lymphoma cell line, where it exhibited excellent potency and selectivity (IC_50_ = 54.1 nM). Recently, Domaille and co-workers demonstrated that DABs react even faster with peroxynitrite.^71^

**Figure 14:**
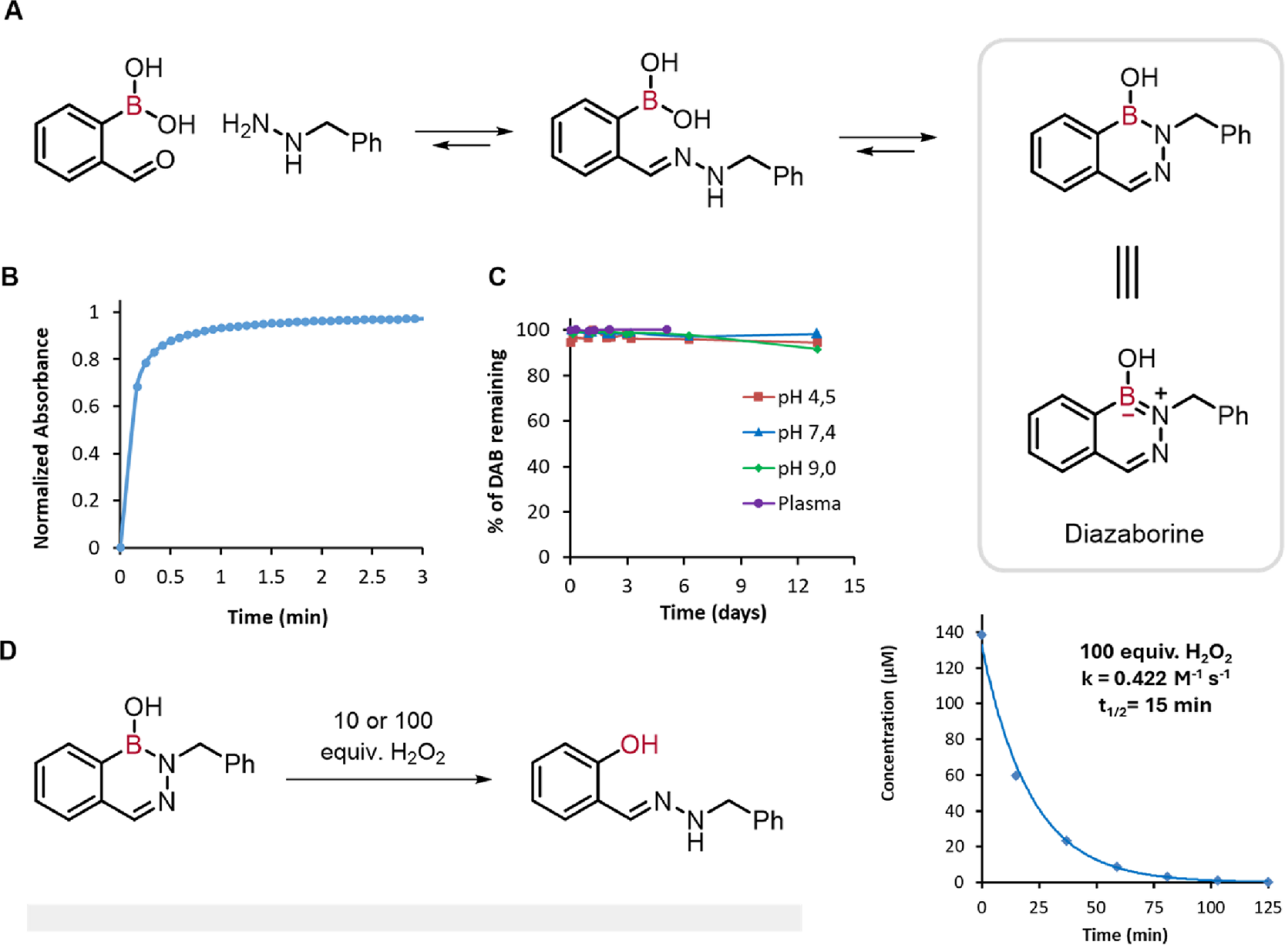
(A) Diazaborine formation mechanism where the formed product is stabilized by a B–N bond. (B) Formation kinetics between 2-FPBA and benzyl hydrazine at pH 7.4. (C) Stability of the diazaborine in different aqueous conditions. (D) DABs are swiftly oxidized to the corresponding phenols in the presence of 10 and 100 equiv of H_2_O_2_.

**Figure 15:**
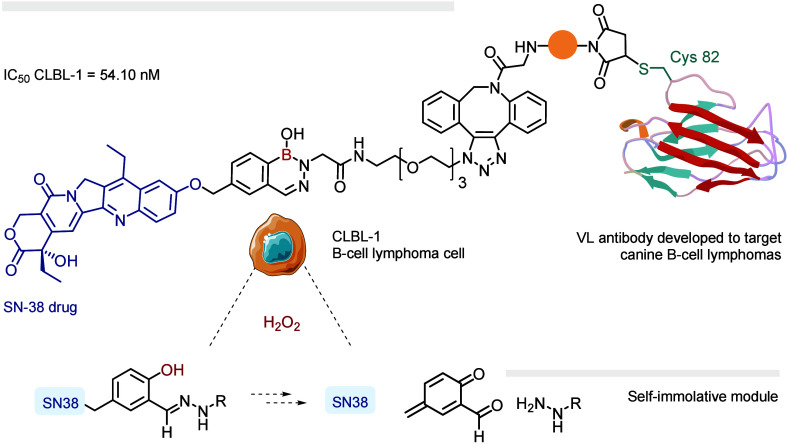
ROS-responsive ADC featuring a cytotoxic SN-38 drug.

## 6 Chemically Induced Responsiveness

### 6.1 Boronated Thiazolidines

Heterogenicity is a major hurdle in the development of therapeutic bioconjugates. In most bioconjugation methodologies available, particularly those targeting natural amino acids, a statistical distribution of modifications is typically observed. This broad range of payload-to-biomolecule ratio is highly undesirable as the pharmacokinetic and toxicity profiles of each species vary accordingly. Therefore, a strong focus has been placed on discovering new site-selective modification technologies that enable precise control over the modification number and location. While this may be achieved through the incorporation of unnatural amino acids, with all the inherent difficulties associated with their preparation, efforts have also been devoted to exploring natural “hotspots” for site selectivity.

A common strategy to achieve homogeneous conjugates is to target *N*-terminal cysteines through their condensation with aldehydes and nitriles to generate thiazolidines.^72^ However, thiazolidine formation displays severe limitations, including the harsh reaction conditions required, an acidic pH of 4–5, long reaction times of up to several days, and the need for several equivalents of reactants. Furthermore, even when successful, the reaction often yields thiazolidine products as mixtures of diastereoisomers.^73^ Therefore, the general incompatibility of these conditions with a wide range of biomolecules has limited the widespread adoption of thiazolidines as a bioconjugation tool. Considering boron’s ability to stabilize ortho condensations, Gao’s group^74^ and our group^2^ independently reported in 2015 the functionalization of the N-terminal cysteine using 2-FPBA. First, an imine is swiftly formed and stabilized by the B–N bond, followed by an intramolecular thiol addition that generates a stable boronated thiazolidine. This reaction was shown to be fast (*k*_2_ = 2.4 × 10^2^ M^–1^ s^–1^), and the product formed was stable in acidic, neutral, and slightly basic pH (pH < 9). Moreover, we demonstrated that the functionalization proceeds efficiently in the presence of structurally diverse adjacent amino acids (e.g., Gly, Tyr, Ser, and Lys), while Gao and co-workers reported that the conjugation reaction tolerates the presence of interfering endogenous molecules such as fructose, serine, lysine, and glutathione. This newly developed technology was successfully used for the site-selective modification of model peptides, including laminin fragments and c-ovalbumin, even in the presence of in-chain cysteines. Importantly, despite its considerable stability at physiological pH, we demonstrated that thiazolidines are swiftly broken down in the presence of hydroxylamines. With this information in hand, we envisioned a strategy for the orthogonal dual modification of the hormone calcitonin, using thiazolidine as a transient protecting group for N-terminal cysteines (Figure 16). A few years later, we also demonstrated that thiazolidines may also be disrupted by the addition of hydrazines, to enable site-selective modification of cysteines.^75^

**Figure 16:**
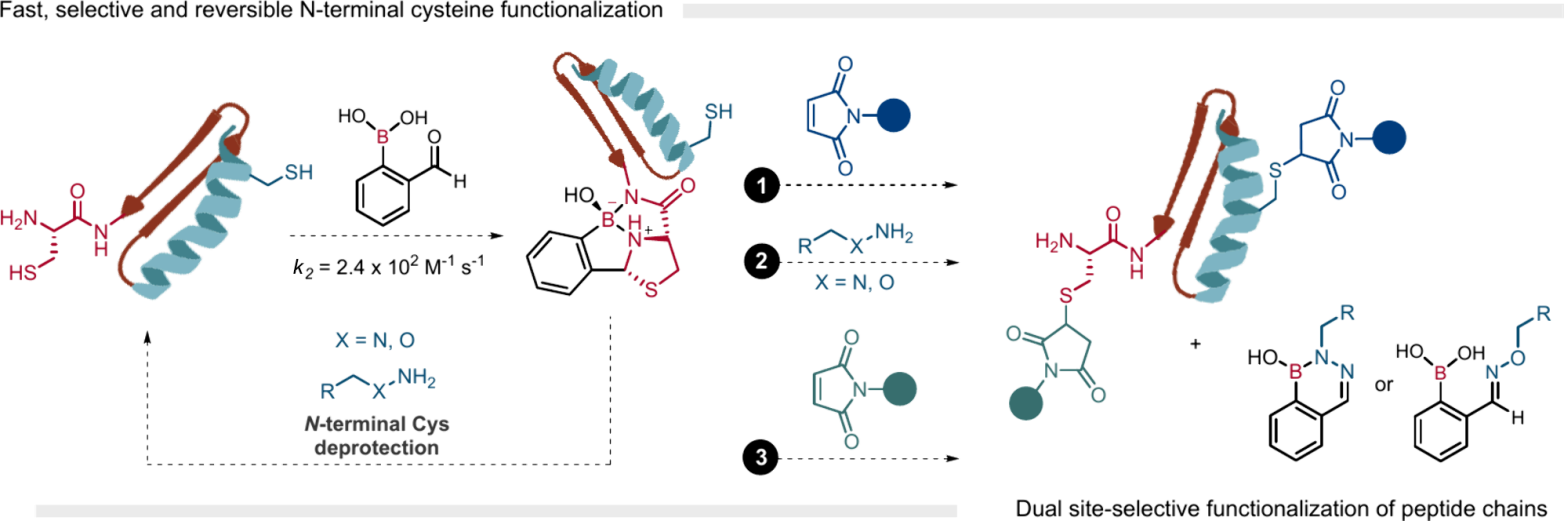
Conjugation of 2-FPBA with different N-terminal cysteines to form a thiazolidine boronate complex. This system may be used to generate dual site-selective bioconjugates

## 7 Conclusions

Boronic acids offer a powerful platform for designing functional molecules that can respond to environmental stimuli, making them indispensable in areas like biosensing, drug delivery, and the creation of smart materials. While challenges remain, particularly concerning their stability and selectivity, ongoing research is actively addressing these hurdles, expanding the potential applications of boronic acids. As a result, they continue to play a pivotal role in the development of next-generation responsive systems with enormous potential across medicine, materials science, and environmental engineering. In this Account, we have brought together various strategies to design BA-based frameworks, focusing on how they respond to both internal and external stimuli (Figure 17). Notably, these scaffolds exhibit dynamic stability profiles that can be fine-tuned for specific uses by carefully selecting ligands and adjusting the boronic acids’ p*K*_a_. One of the most exciting aspects of boronic acids is their reactivity, biocompatibility, and mild Lewis acidity under physiological conditions, which allow for precise modifications of functional groups in complex systems. This occurs through the reversible complexation of uncharged sp^2^ BAs with endogenous Lewis bases, such as nitrogen, oxygen, sulfur, or phosphorus atoms in biomolecules, leading to the formation of negatively charged sp^3^ boronates. In many cases, the boron center serves as a template for ligand binding or enhances the reactivity of nearby functional groups, highlighting its versatility as a building block. However, the potential of boronic acids extends far beyond their role as simple framework assemblers. Their stability is highly sensitive to environmental changes, such as pH shifts or the presence of endogenous molecules, opening possibilities for designing controlled, reversible systems. In this Account, we also focus on how BAs can be used to develop materials with built-in mechanisms that respond precisely to chemical stimuli like pH changes, carbohydrates, GSH, reactive oxygen species, and other external triggers. This ability to respond to specific stimuli is particularly valuable in developing precision therapies for complex diseases such as cancer, neurodegeneration, and inflammation, where these chemical triggers often play key roles. We highlight contributions from other researchers who have explored complementary methods, further emphasizing the broad utility of boronic acids. Their unique ability to form diverse frameworks with precise, reversible properties has made them essential tools for probing biological and chemical processes. While their success in applications like targeted drug delivery, biomolecule modification, biological labeling, separation, and sensing is well established, challenges remain in translating these successes from in vitro experiments to clinical settings. In particular, the high reactivity and low oxidative stability of boronic acids necessitate careful, application-specific design strategies. Despite these challenges, boronic acids have come a long way. What started as simple sacrificial building blocks in Pd-catalyzed cross-couplings have evolved into a key element of modern chemical biology and medicinal chemistry. While there’s still work to be done, the successes outlined in this Account point to a bright future for this simple yet incredibly versatile functional group.

**Figure 17:**
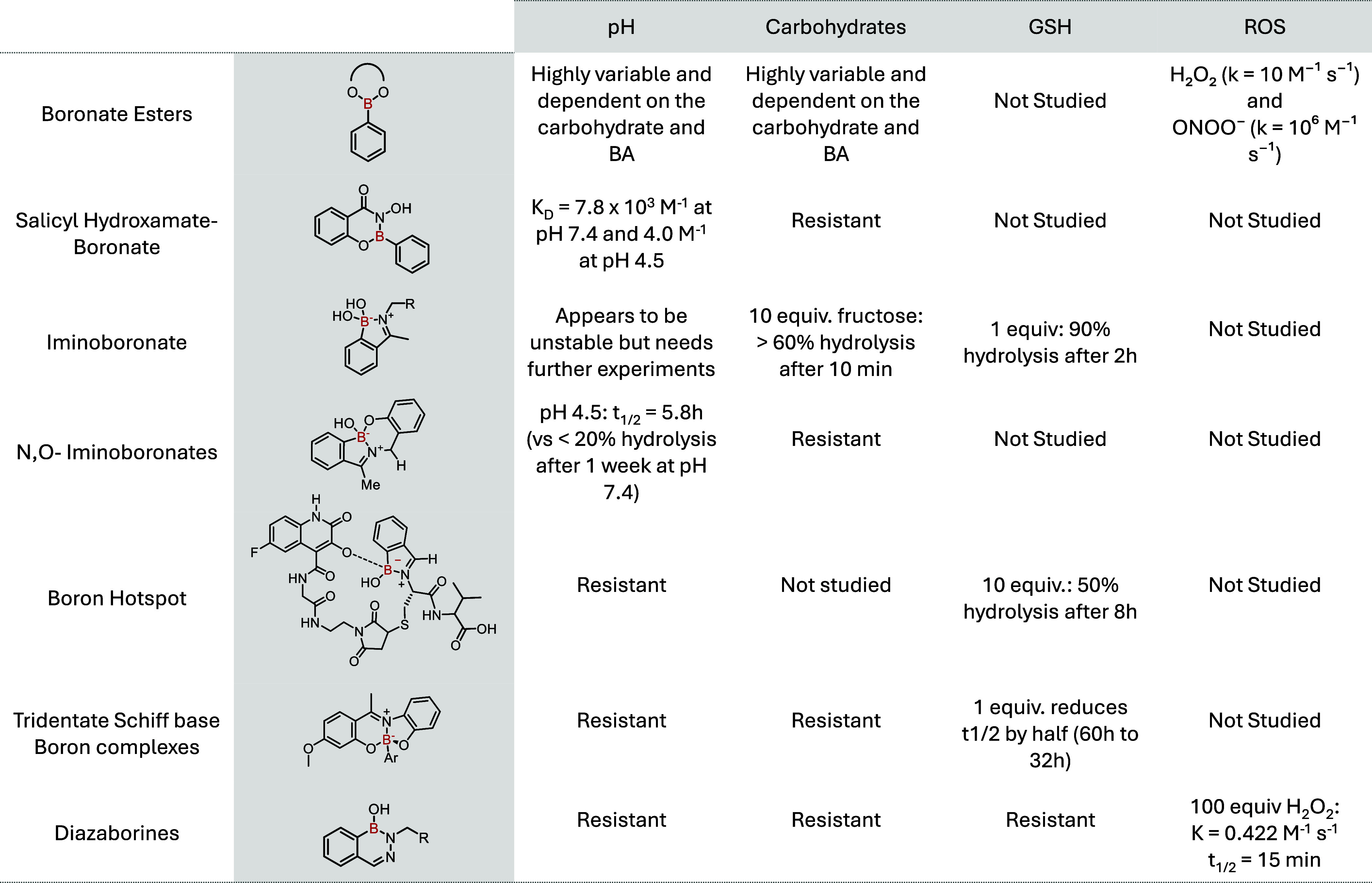
Table summarizing the chemical structures detailed in this Account and their susceptibility/resistance to different endogenous stimuli.
